# Preventing type 2 diabetes mellitus in Qatar by reducing obesity, smoking, and physical inactivity: mathematical modeling analyses

**DOI:** 10.1186/s12963-019-0200-1

**Published:** 2019-12-30

**Authors:** Susanne F. Awad, Martin O’Flaherty, Katie G. El-Nahas, Abdulla O. Al-Hamaq, Julia A. Critchley, Laith J. Abu-Raddad

**Affiliations:** 1 Infectious Disease Epidemiology Group, Weill Cornell Medicine – Qatar, Qatar Foundation - Education City, P.O. Box 24144, Doha, Qatar; 2 Population Health Research Institute, St George’s, University of London, London, UK; 3 Division of Public Health, University of Liverpool, Liverpool, UK; 4 Qatar Diabetes Association, Doha, Qatar; 5 Department of Healthcare Policy and Research, Weill Cornell Medicine, Cornell University, New York, NY USA; 6 College of Health and Life Sciences, Hamad bin Khalifa University, Doha, Qatar

**Keywords:** Noncommunicable disease, type 2 diabetes mellitus, Obesity, Risk factors, Prevention, Mathematical modeling, Middle East and North Africa

## Abstract

**Background:**

The aim of this study was to estimate the impact of reducing the prevalence of obesity, smoking, and physical inactivity, and introducing physical activity as an explicit intervention, on the burden of type 2 diabetes mellitus (T2DM), using Qatar as an example.

**Methods:**

A population-level mathematical model was adapted and expanded. The model was stratified by sex, age group, risk factor status, T2DM status, and intervention status, and parameterized by nationally representative data. Modeled interventions were introduced in 2016, reached targeted level by 2031, and then maintained up to 2050. Diverse intervention scenarios were assessed and compared with a counter-factual no intervention baseline scenario.

**Results:**

T2DM prevalence increased from 16.7% in 2016 to 24.0% in 2050 in the baseline scenario. By 2050, through halting the rise or reducing obesity prevalence by 10–50%, T2DM prevalence was reduced by 7.8–33.7%, incidence by 8.4–38.9%, and related deaths by 2.1–13.2%. For smoking, through halting the rise or reducing smoking prevalence by 10–50%, T2DM prevalence was reduced by 0.5–2.8%, incidence by 0.5–3.2%, and related deaths by 0.1–0.7%. For physical inactivity, through halting the rise or reducing physical inactivity prevalence by 10–50%, T2DM prevalence was reduced by 0.5–6.9%, incidence by 0.5–7.9%, and related deaths by 0.2–2.8%. Introduction of physical activity with varying intensity at 25% coverage reduced T2DM prevalence by 3.3–9.2%, incidence by 4.2–11.5%, and related deaths by 1.9–5.2%.

**Conclusions:**

Major reductions in T2DM incidence could be accomplished by reducing obesity, while modest reductions could be accomplished by reducing smoking and physical inactivity, or by introducing physical activity as an intervention.

## Background

About 90% of individuals with diabetes mellitus have type 2 diabetes (T2DM), a largely preventable disease that is influenced by several modifiable risk factors such as obesity, active smoking, physical inactivity, and unhealthy diet [1–7]. In 2017, diabetes was estimated to cause 6.7 million deaths, becoming the eighth leading cause of death worldwide [8]. If no further action is taken, the epidemic of diabetes is projected to increase from 425 million in 2017 to 629 million by 2045 [9].

Strategies to reduce and control the modifiable risk factors for T2DM have been previously demonstrated and utilized [10–15], and could reduce the burden of T2DM by averting new cases and preventing premature deaths. The World Health Organization (WHO) recommends a comprehensive global action plan for the prevention and control of noncommunicable diseases (NCDs) including diabetes [16]. The action plan provides a set of voluntary global policy recommendations to attain national health targets for 2025. These targets include a 25% relative reduction in premature mortality from NCDs between 2010 and 2025, halting the rise of both diabetes and obesity, and reducing the prevalence of three behavioral risk factors for NCDs: tobacco use (by 30%), unhealthy diet (by 30%), and physical inactivity (by 10%) [16]. However, limited studies have quantitatively assessed the country-specific effects of changes in these risk factors on T2DM burden [17–19].

Diabetes prevalence in Qatar is twice the global prevalence [9]. In a recent modeling study, the prevalence of T2DM among Qataris was projected to increase from 17% in 2012 to at least 24% by 2050 [20]. Obesity was found to be the principal risk factor explaining nearly two-thirds of T2DM incidence [20]. National T2DM health expenditure was projected to account for up to 32% of Qatar’s total health expenditure by 2050 [20].

With the urgent need for effective public health strategies to reduce the burden of T2DM in Qatar, the objective of this study was to evaluate the impact of several generic intervention scenarios aimed at reducing the prevalence of T2DM-related risk factors, on T2DM incident cases and related deaths. While this study is an application for Qatar, as an illustrative example, the methodology and approach developed here can be applied to other countries. Generally, the present study demonstrates the impact of reducing T2DM-related risk factors in countries with high T2DM prevalence, and where specific guidelines to overcome T2DM challenges are not yet in place.

## Methods

We extended a recently developed T2DM age-structured mathematical model by including intervention compartments and their dynamics, representing individuals in the intervention groups, to investigate the impact of different generic intervention scenarios on T2DM epidemiology. Details on the original model can be found in Awad et al. [20]. Briefly, the model was a population-based deterministic compartmental model accounting for the epidemiological dynamics of T2DM including key risk factors and their overlap. The model was expressed by a set of differential equations stratifying the population according to sex, age, risk factor (obesity, smoking, and physical inactivity) status, and T2DM status. Obesity was defined as BMI > 30 kg/m^2^ across all age groups. The population was further disaggregated by intervention status—every susceptible state (no T2DM) in the model was categorized according to the physical activity intervention status (see Additional file 1: Figure S1). Further details on the model structure and assumptions can be found in see Additional file 1: Text S1 and Table S1.

The model was parameterized using epidemiological and natural history data, listed in Additional file 1: Table S2. Further discussion of parameter values is provided in Awad et al. [20]. The model was fitted to sex- and age-specific T2DM, obesity, smoking, and physical inactivity prevalence data for Qataris using a nonlinear least-square fitting method [21] and programmed in MATLAB 2015a [22]. The model’s MATLAB codes can be obtained by contacting the authors.

### T2DM intervention scenarios targeting risk factors

The observation period for all intervention scenarios was 2016–2050. The starting year (2016) was chosen based on the year Qatar launched the National Diabetes Strategy [23]. For all modeled scenarios, we assumed that the intervention was introduced in 2016 among Qataris aged 15–64 years and lasted for 15 years. This 15-year time horizon was based on WHO’s recommendation for the Global Action Plan for NCDs [16]. The prevalence of each targeted risk factor achieved by 2031 was then maintained between 2032 and 2050.

The modeled intervention scenarios targeted only the susceptible (diabetes free) population, since the study scope was to explore the impact of health interventions on *preventing* T2DM incident cases and related deaths, not on reducing further health complications associated with T2DM.

The implications of the modeled intervention scenarios were assessed through epidemiologic measures. For each modeled scenario, the primary outcome of interest was the predicted trend in T2DM prevalence among adult Qataris aged 15–64 years between 2016 and 2050. The predicted T2DM prevalence in the presence of the intervention was then compared with the prevalence in a counter-factual (baseline) scenario with no intervention, as reported in Awad et al. [20]. In this baseline scenario, the prediction for T2DM prevalence between 2016 and 2050 was based on the assumption that the age-specific prevalence of risk factors remained the same between 2016 and 2050 [20]. Accordingly, the trends in risk factors depended only on the demographic structure of the population that is on the population distribution across the different age groups. Of note that the average age of the population is increasing steadily [20]—the Qatari population is currently relatively young, but is expected to age rapidly over time [20, 24].

We also estimated the number of T2DM cases averted and reduction in T2DM-related deaths by comparing the cumulative population-level T2DM incidence and deaths in the presence of the intervention with the baseline scenario.

The impact of the modeled scenarios on T2DM cases and deaths was evaluated in two time horizons: an intermediate-term (2016–2031) and a long-term time horizon (2016–2050). The modeled intervention scenarios are summarized in Table 1.

**Table 1:** Impact of different generic intervention-for-prevention scenarios on the cases and deaths of type 2 diabetes mellitus (T2DM) among Qataris between 2016 and 2031

Modelled scenario	*T2DM prevalence* (2031)	Relative reduction in *T2DM prevalence*	# of T2DM cases averted (2016-2031)	Proportion of T2DM cases averted	# of T2DM-related deaths averted (2016-2031)	Proportion of T2DM-related deaths averted	*T2DM prevalence* (2050)	Relative reduction in *T2DM prevalence*	# of T2DM cases averted (2016-2050)	Proportion of T2DM cases averted	# of T2DM-related deaths averted (2016-2050)	Proportion of T2DM-related deaths averted
***Baseline with no intervention***	**18.6%**	**-**	**-**	**-**	**-**	**-**	**24.0%**	**-**	**-**	**-**	**-**	**-**
*Impact of the World Health Organization Global Action Plan for Noncommunicable Diseases* (Fig. 1)												
Halt *P*_*O*_, reduce *P*_*S*_ by 30% and reduce *P*_*PIA*_ by 10%	17.7%	4.7%	2148	7.0%	28	0.7%	21.0%	12.0%	9147	13.2%	383	3.2%
*Impact of reducing (by 2031) the prevalence of obesity (P*_*O*_*) in the population* (Fig. 2)												
Halt *P*_*O*_ at 41.4%	18.2%	2.0%	958	3.1%	15	0.4%	22.0%	7.8%	5,828	8.4%	247	2.1%
Reduce *P*_*O*_ by 10% (*P*_*O*_=37.3% in 2031)	17.7%	5.1%	2,359	7.7%	38	0.9%	20.8%	12.8%	9,914	14.3%	508	4.3%
Reduce *P*_*O*_ by 20% (*P*_*O*_=33.1% in 2031)	17.1%	8.0%	3,696	12.1%	60	1.4%	19.6%	17.8%	14,000	20.2%	753	6.4%
Reduce *P*_*O*_ by 30% (*P*_*O*_=29.0% in 2031)	16.4%	11.7%	5,409	17.7%	90	2.1%	18.2%	23.8%	18,848	27.2%	1,050	8.9%
Reduce *P*_*O*_ by 40% (*P*_*O*_*=*24.8% in 2031)	15.8%	15.0%	6,945	22.7%	117	2.8%	17.1%	28.4%	22,646	32.7%	1,297	11.0%
Reduce *P*_*O*_ by 50% (*P*_*O*_=20.7% in 2031)	15.1%	18.8%	8,688	28.4%	151	3.6%	15.8%	33.7%	26,930	38.9%	1,564	13.2%
*Impact of reducing (by 2031) the prevalence of smoking (P*_*S*_*) in the population* (Additional file 1: Figure S3)												
Halt *P*_*S*_ at 16.2%	18.6%	0.2%	79	0.3%	1	0.02%	23.8%	0.5%	321	0.5%	11	0.1%
Reduce *P*_*S*_ by 10% (*P*_*S*_=14.6% in 2031)	18.5%	0.5%	243	0.7%	3	0.1%	23.6%	1.2%	931	1.3%	33	0.3%
Reduce *P*_*S*_ by 20% (*P*_*S*_=13.0% in 2031)	18.5%	0.7%	343	1.1%	4	0.1%	23.5%	1.5%	1,222	1.8%	45	0.4%
Reduce *P*_*S*_ by 30% (*P*_*S*_=11.3% in 2031)	18.4%	1.0%	458	1.5%	5	0.1%	23.4%	2.0%	1,559	2.3%	59	0.5%
Reduce *P*_*S*_ by 40% (*P*_*S*_=9.7% in 2031)	18.4%	1.3%	608	2.0%	7	0.2%	23.3%	2.5%	1,999	2.9%	77	0.7%
Reduce *P*_*S*_ by 50% (*P*_*S*_=8.1% in 2031)	18.3%	1.5%	693	2.3%	9	0.2%	23.2%	2.8%	2,184	3.2%	86	0.7%
*Impact of reducing (by 2031) the prevalence of physical inactivity (P*_*PIA*_*) in the population* (Additional file 1: Figure S4)												
Halt *P*_*PIA*_ at 46.0%	18.6%	0.2%	111	0.4%	2	0.05%	23.7%	0.5%	350	0.5%	22	0.2%
Reduce *P*_*PIA*_ by 10% (*P*_*PIA*_=41.4% in 2031)	18.4%	1.0%	488	1.6%	9	0.2%	23.3%	2.5%	1,969	2.8%	106	0.9%
Reduce *P*_*PIA*_ by 20% (*P*_*PIA*_=36.8% in 2031)	18.3%	1.5%	739	2.4%	14	0.3%	23.0%	3.6%	2,854	4.1%	157	1.3%
Reduce *P*_*PIA*_ by 30% (*P*_*PIA*_=32.2% in 2031)	18.2%	2.2%	1,029	3.4%	19	0.5%	22.7%	4.7%	3,691	5.3%	210	1.8%
Reduce *P*_*PIA*_ by 40% (*P*_*PIA*_=27.6% in 2031)	18.1%	2.9%	1,365	4.5%	26	0.6%	22.5%	5.8%	4,614	6.7%	270	2.3%
Reduce *P*_*PIA*_ by 50% (*P*_*PIA*_=23.0% in 2031)	17.9%	3.7%	1,744	5.7%	34	0.8%	22.2%	6.9%	5,482	7.9%	332	2.8%

*P*_*O*_: obesity prevalence; *P*_*S*_: smoking prevalence; *P*_*PIA*_: physical inactivity prevalence.

#### Impact of WHO’s Global Action Plan for NCDs

In this modeled intervention scenario, by implementing the WHO’s Global Action Plan for NCDs [16], we investigated the impact of changing the prevalence of obesity, smoking, and physical inactivity simultaneously. Based on the 2016 prevalence for each risk factor, we halted the prevalence of obesity and reduced (linearly) the prevalence of smoking and physical inactivity by 30% and 10%, respectively, between 2016 and 2031. Accordingly, by 2031, the prevalence of obesity, smoking, and physical inactivity reached 41.4%, 11.3%, and 41.4%, respectively.

#### Impact of reducing the prevalence of T2DM-related risk factors

In these modeled intervention scenarios, we focused on three main risk factors for T2DM: obesity, smoking, and physical inactivity. We generated three sets of scenarios where the prevalence of each risk factor was separately reduced by various proportions between 2016 and 2031. Based on the 2016 prevalence for each risk factor in the Qatari population, we assumed a relative reduction in risk factor prevalence of 10%, 20%, 30%, 40%, and 50% by 2031.

Accordingly, for the first set of scenarios, the prevalence of obesity was reduced from 41.4% in 2016 [20] to 37.3%, 33.1%, 29.0%, 24.8%, and 20.7% by 2031, respectively. For the second set of scenarios, the prevalence of smoking was reduced from 16.2% in 2016 [20] to 14.6%, 13.0%, 11.3%, 9.7%, and 8.1% by 2031, respectively. For the third set of scenarios, the prevalence of physical inactivity was reduced from 46.0% in 2016 [20] to 41.4%, 36.8%, 32.2%, 27.6%, and 23.0% by 2031, respectively.

We also investigated the impact of simultaneously reducing the prevalence of obesity, smoking, and physical inactivity by 50% by 2031. Accordingly, by 2031, the prevalence of obesity, smoking, and physical inactivity reached 20.7%, 8.1%, and 23.0%, respectively, and then was maintained at these levels between 2032 and 2050.

In an additional analysis, given the prominent role of obesity in driving T2DM incidence in Qatar [20], we explored the impact of varying the duration of scale-up for an intervention that reduces obesity prevalence by 40%. The different scale-up durations ranged between 5 to 20 years.

#### Impact of introducing different intensities of physical activity as an intervention

In this modeled intervention scenario, physical activity was introduced as an *explicit* intervention in the population with varying levels of intensity—as opposed to merely reducing the prevalence of physical inactivity, as in the above-described scenarios. Physical activity as an intervention was assumed to affect the population by reducing the risk of developing T2DM among susceptible (diabetes free) individuals (e.g., healthy, obese, smoker, or physically inactive) in the intervention group. For instance, individuals in the obese state that become physically active will have a lower risk of developing T2DM in comparison with obese individuals that are not physically active. The relative risks of developing T2DM were incorporated assuming independence of risk factors.

The impact of interventional physical activity on reducing the risk of developing T2DM was assumed to change with five different levels of physical activity. We assumed that the relative risk for developing T2DM (relative to the baseline of no intervention) was 0.61 for vigorous activity, 0.66 for low-intensity activity, 0.68 for moderate activity, 0.74 for leisure-time activity, and 0.85 for walking. These relative risks were based on a recent systematic review and meta-analysis [25].

We assumed that interventional physical activity coverage reached 25% in the population by 2031. The 25% coverage was based on evidence reporting an increase in the proportion of the population that became physically active in intervention strategies that created and/or enhanced access to places for physical activity combined with informational outreach activities [26].

In an additional analysis, we investigated the impact of simultaneously reducing the prevalence of obesity, smoking, and physical inactivity by 50% by 2031, combined with 25% coverage of vigorous physical activity.

## Results

Additional file 1: Figure S2 shows the model-projected demographics of the Qatari population. The size of the 15–64-year-old population was estimated at 144,066; 201,499; and 221,627; in 2016, 2031, and 2050, respectively. In the baseline scenario with no intervention, the total number of T2DM cases was estimated at 24,043; 37,104; and 53,207; in 2016, 2031, and 2050, respectively, with the corresponding prevalence being 16.7%, 18.3%, and 24.0%. Similarly for these years, the total number of T2DM-related deaths was estimated at 206, 320, and 529, respectively, with the corresponding case fatality rate being 8.6, 8.6, and 9.9 per 1000 persons per year.

### Impact of WHO’s Global Action Plan for NCDs

**Fig. 1:**
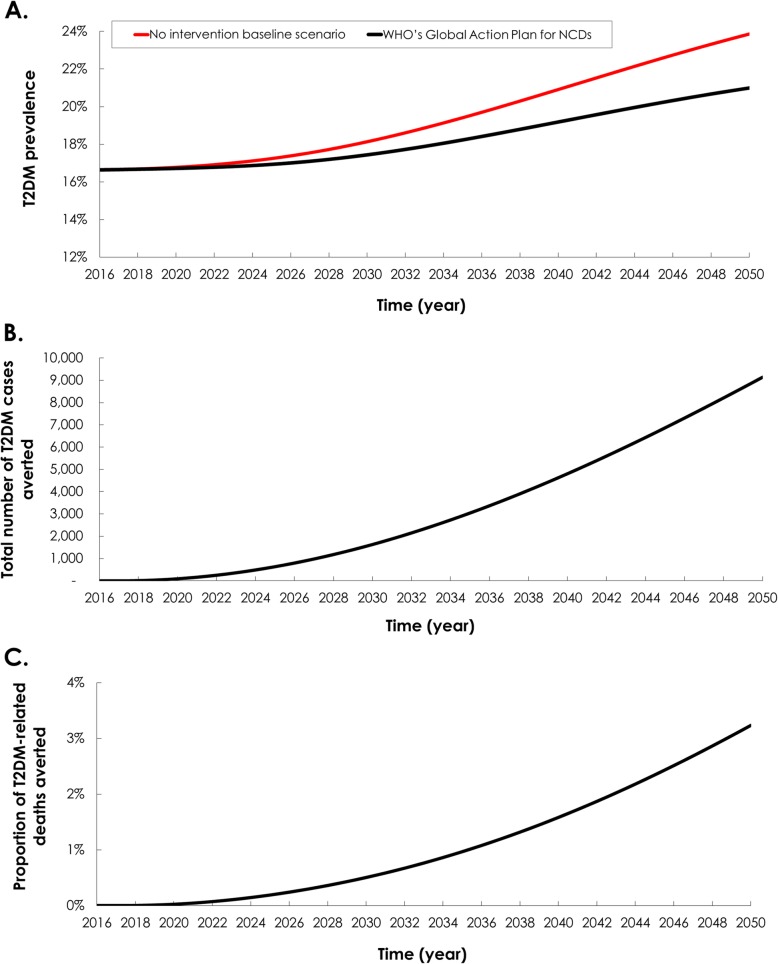
Epidemiologic impact of reducing the prevalence of obesity, smoking, and physical inactivity according to the World Health Organization Global Action Plan for Noncommunicable Diseases (NCDs). The figure shows the **a** projection of type 2 diabetes mellitus (T2DM) prevalence among 15–64 years old Qataris, **b** number of T2DM cases averted, and **c** proportion of T2DM-related deaths averted. The red curve in panel a shows the prediction of T2DM prevalence in the no intervention scenario (baseline scenario)

The predicted impact on T2DM burden of reducing the prevalence of T2DM risk factors to the levels recommended by the WHO Global Action Plan for NCDs [16] is described in Table 1 and Fig. 1. In the baseline scenario with no intervention, T2DM prevalence increased from 16.7% in 2016 to 18.6% by 2031. In the intervention scenario, T2DM prevalence reached 17.7% by 2031—4.7% lower than in the baseline scenario. By 2050, T2DM prevalence reached 24.0% in the baseline scenario and 21.0% in the intervention scenario—12.0% lower than in the baseline scenario.

The model estimated that 2148 T2DM cases could be averted between 2016 and 2031 in the intervention scenario (i.e., 7.0% of new T2DM incident cases; Table 1 and Fig. 1b). Between 2016 and 2050, the number of T2DM cases averted reached 9,147—13.2% of new T2DM incident cases. Comparing the intervention scenario to the baseline scenario, 28 T2DM-related deaths could be prevented between 2016 and 2031 (i.e., 0.7% of all T2DM-related deaths), while 383 T2DM-related deaths could be prevented between 2016 and 2050—3.2% of all T2DM-related deaths (Table 1 and Fig. 1c).

### Impact of reducing the prevalence of each of the T2DM-related risk factors

**Fig. 2:**
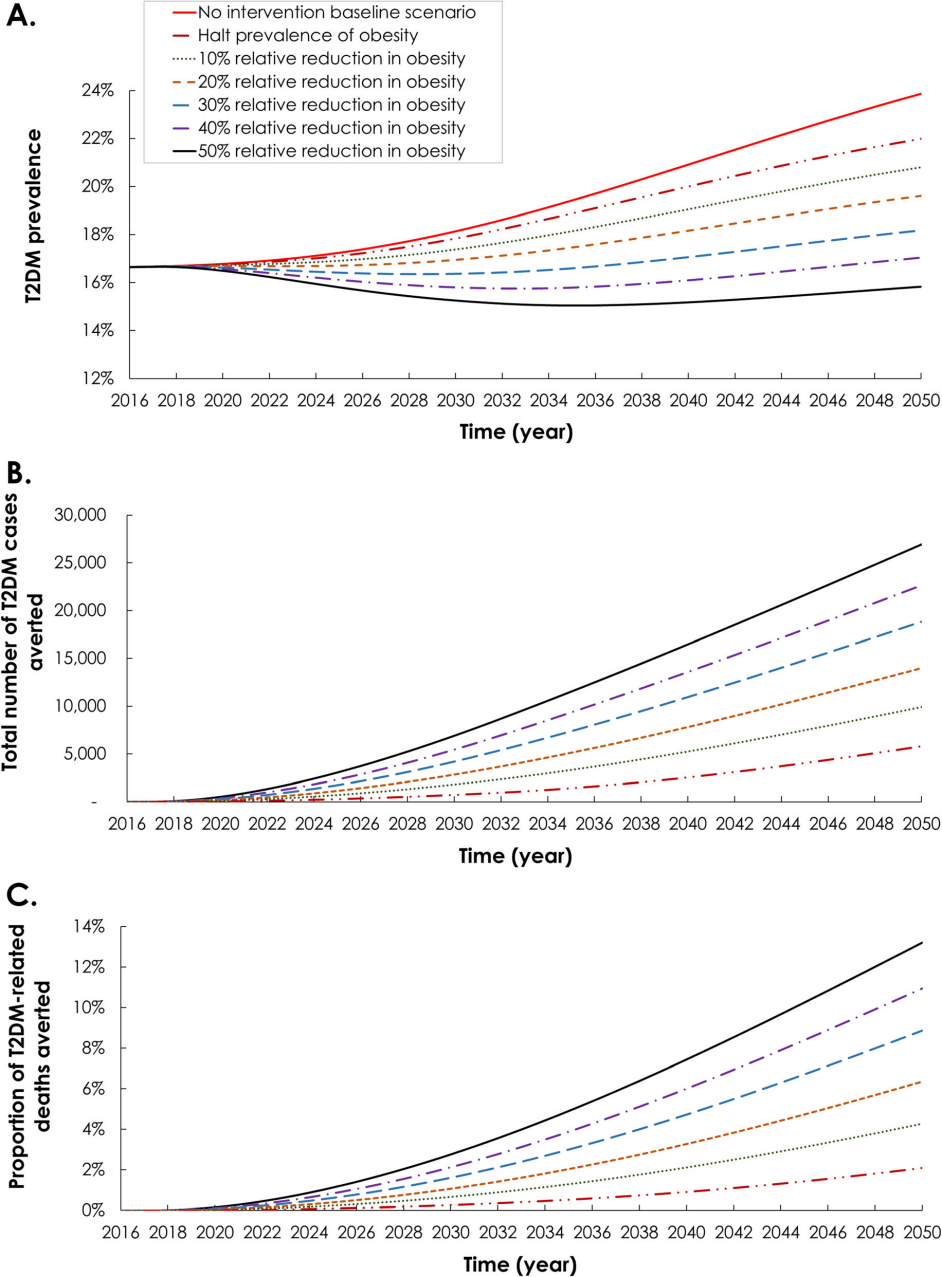
Epidemiologic impact of reducing the prevalence of obesity among Qataris 15–64 years of age. The figure shows the **a** projection of type 2 diabetes mellitus (T2DM) prevalence, **b** number of T2DM cases averted, and **c** proportion of T2DM-related deaths averted. The solid red curve in panel **a** shows the prediction of T2DM prevalence in the no intervention scenario (baseline scenario)

Figure 2 and Additional file 1: Figures S3 and S4 show six modeled intervention scenarios for each of the T2DM-related risk factors: obesity, smoking, and physical inactivity. These scenarios assessed the impact on T2DM burden of reducing the prevalence of each risk factor by 2031, and keeping it constant thereafter. The scenarios ranged between halting the prevalence of the risk factor at its 2016 level, to reducing it by 50% relative to its 2016 level.

#### Reducing obesity prevalence

Across the six obesity reduction scenarios, by 2031, obesity prevalence is reduced from 41.4% down to 20.7%. Accordingly, T2DM prevalence (absolute level) ranged from 18.2% down to 15.1%; 2.0–18.8% lower than in the baseline scenario (Table 1 and Fig. 2). The number of averted T2DM cases ranged from 958–8,688—3.1–28.4% of new T2DM incident cases. In total, 15–151 T2DM-related deaths could be prevented—0.4–3.6% of all T2DM-related deaths.

Similarly, by 2050, T2DM prevalence ranged from 22.0% down to 15.8%; 7.8–33.7% lower than in the baseline scenario (Table 1 and Fig. 2). The number of averted T2DM cases ranged from 5828–26,930—8.4–38.9% of new T2DM incident cases. In total, 247–564 T2DM-related deaths could be prevented—2.1–13.2% of all T2DM-related deaths.

In an additional analysis, reducing obesity prevalence by 40% over 5, 10, 15, and 20 years, respectively, 7963; 6939; 6094; and 5642 T2DM incident cases were averted by 2031, and 24,479; 23,473; 22,570; and 21,521 T2DM cases by 2050 (see Additional file 1: Figure S5).

#### Reducing smoking prevalence

Across the six smoking reduction scenarios, by 2031, smoking prevalence is reduced from 16.2% down to 8.1%. Accordingly, T2DM prevalence ranged between 18.6% down to 18.3%; 0.2–1.5% lower than in the baseline scenario (Table 1 and see Additional file 1: Figure S3). The number of T2DM cases averted ranged from 79–693—0.3–2.3% of new T2DM incident cases. In total, 1–9 T2DM-related deaths could be prevented—0.02–0.2% of all T2DM-related deaths.

Similarly, by 2050, T2DM prevalence ranged between 23.8% down to 23.2%; 0.5–2.8% lower than in the baseline scenario (Table 1 and see Additional file 1: Figure S3). The number of T2DM cases averted ranged from 321–2184—0.5–3.2% of new T2DM incident cases. In total, 11–86 T2DM-related deaths would be prevented—0.1–0.7% of total T2DM-related deaths.

#### Reducing physical inactivity prevalence

Across the six physical inactivity reduction scenarios, by 2031, physical inactivity prevalence is reduced from 46.0% down to 23.0%. Accordingly, T2DM prevalence ranged between 18.6% down to 17.9%; 0.2–3.7% lower than in the baseline scenario (Table 1 and see Additional file 1: Figure S4). The number of averted T2DM cases ranged from 111–1744—0.4–5.7% of new T2DM incident cases. In total, 2–34 T2DM-related deaths could be prevented—0.05–0.8% of all T2DM-related deaths.

Similarly, by 2050, T2DM prevalence ranged between 23.7% down to 22.2%; 0.5–6.9% lower than in the baseline scenario (Table 1 and see Additional file 1: Figure S4). The number of averted T2DM cases ranged from 350–5482—0.5–7.9% of new T2DM incident cases. In total, 22–332 T2DM-related deaths could be prevented—0.2–2.8% of all T2DM-related deaths.

#### Simultaneous reduction of risk factors

In the scenario of simultaneously reducing the prevalence of obesity, smoking, and physical inactivity by 50%, T2DM prevalence reached 14.9% by 2031—20.0% lower than in the baseline scenario (see Additional file 1: Figure S6A). By 2050, T2DM prevalence reached 15.0% in the intervention scenario—37.0% lower than in the baseline scenario.

### Impact of introducing different intensities of physical activity as an intervention

**Fig. 3:**
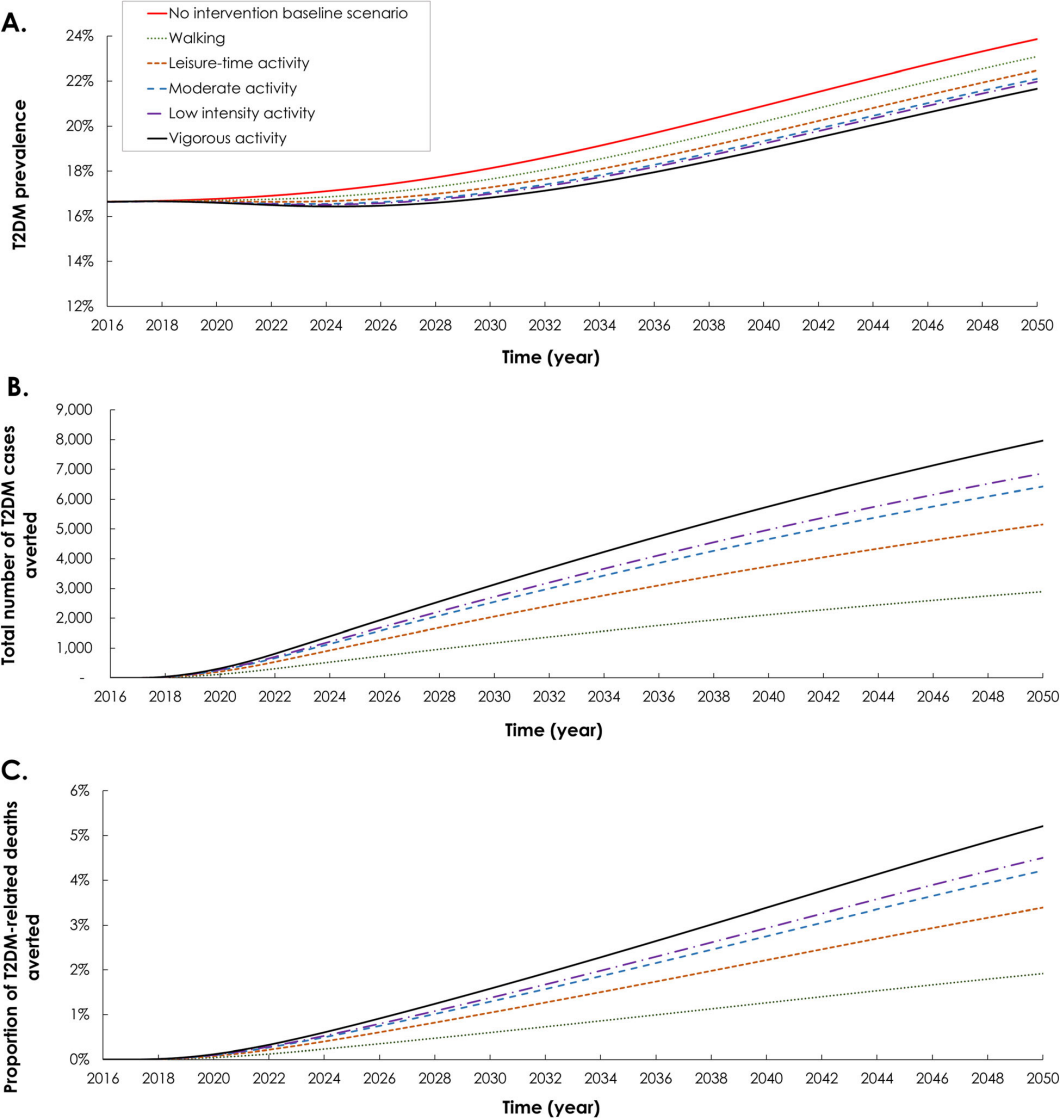
Epidemiologic impact of increasing the coverage of various levels of physical activity as an intervention among Qataris 15–64 years of age. The intervention coverage was increased steadily up to 25% by 2031, and then kept constant at this level up to 2050. The figure shows the **a** projection of type 2 diabetes mellitus (T2DM) prevalence, **b** number of T2DM cases averted, and **c** proportion of T2DM-related deaths averted. The solid red curve in panel **a** shows the baseline prediction of T2DM prevalence of the no intervention scenario (baseline scenario)

Figure 3 shows the five modeled scenarios that assessed the impact on T2DM burden of increasing the coverage of physical activity as an intervention. The intervention coverage was increased steadily up to 25% by 2031 and then kept constant at this level up to 2050. Though coverage was set at 25%, the scenarios varied in the type and intensity of physical activity being introduced as an intervention, from walking up to vigorous activity.

Across these scenarios, by 2031, T2DM prevalence ranged between 18.1% down to 17.1%; 2.9–7.9% lower than in the baseline scenario (Table 1 and Fig. 3). The number of averted T2DM cases ranged from 1375–3689—4.5–12.0% of new T2DM incident cases. In total, 31–81 T2DM-related deaths could be prevented—0.7–1.9% of all T2DM-related deaths.

Similarly, by 2050, T2DM prevalence ranged between 23.1% down to 21.7%; 3.3–9.2% lower than in the baseline scenario (Table 1 and Fig. 3). The number of averted T2DM cases ranged from 2892–7966—4.2–11.5% of new T2DM incident cases. In total, 227–616 T2DM-related deaths could be prevented—1.9–5.2% of all T2DM-related deaths.

In the scenario simultaneously reducing the prevalence of obesity, smoking, and physical inactivity by 50%, combined with a 25% coverage of vigorous physical activity in the population, T2DM prevalence reached 13.6% by 2031—27.1% lower than in the baseline scenario. By 2050, T2DM prevalence reached 13.2% in the intervention scenario—44.5% lower than in the baseline scenario (see Additional file 1: Figure S6B).

## Discussion

Using an analytical approach, we investigated the impact of several population-based interventions on reducing the burden of T2DM in Qatar. By targeting and reducing the prevalence of three key T2DM risk factors, namely obesity, smoking, and physical inactivity, up to 46% of future T2DM cases and 14% of T2DM-related deaths can be averted by 2050. While implementing the WHO’s Global Action Plan for NCDs was predicted to avert 13% of future T2DM cases and 3% of T2DM-related deaths by 2050, this impact can only be seen as modest in context of the large and growing T2DM epidemic in Qatar. These findings, therefore, highlight the relevance of the present study in a context in which Qatar has recently launched the National Diabetes Strategy, urging the identification and development of T2DM interventions [23].

The study results highlight that major reductions in T2DM incidence can only be achieved by a marked reduction in obesity prevalence across teens and adults. Reducing obesity prevalence by 50% can avert 39% of future T2DM cases and 13% of T2DM-related deaths by 2050. Reducing the prevalence of smoking and physical inactivity, each also by 50%, can avert only 3% and 8% of T2DM cases, respectively, and 1% and 3% of T2DM-related deaths. Introduction of physical activity as an intervention, with varying levels of intensity and at a coverage of 25%, can also only avert up to 12% of T2DM cases and 5% of T2DM-related deaths by 2050. However, the impact of simultaneously reducing obesity, smoking, and physical inactivity levels combined with increasing physical activity as an intervention can amplify the impact on T2DM, and even reverse the growth in T2DM prevalence (see Additional file 1: Figure S6).

These findings should not be surprising given that obesity is the main driver of T2DM incidence in Qatar, as demonstrated recently [20]. In 2016, 58% of T2DM incidence was predicted to be attributed to obesity as a risk factor, and by 2050, 66% of incidence was attributed to obesity. While controlling the large and expanding T2DM epidemic requires targeting the different T2DM’s key risk factors, the results of the present analyses demonstrate that the core focus should be on targeting obesity. It will be virtually impossible to make a major dent on this epidemic without an aggressive intervention against this risk factor.

Despite the potential impact of targeting these key risk factors, the results also highlight the challenges in halting or reversing the expanding T2DM epidemic in Qatar. Even assuming constant age-specific prevalence for the risk factors between 2016 and 2050, T2DM prevalence was projected to increase by 43%, driven by the fairly rapid aging of the Qatari population [20]. In all intervention scenarios, apart from targeting obesity, T2DM prevalence was projected to increase despite the intervention’s impact in reducing T2DM incidence. Even when targeting obesity, T2DM prevalence in 2050 will be higher than that in 2016 unless obesity can be reduced by over 40% by 2031, which seems unlikely (Table 1 and Fig. 2). Faster scale-up of interventions may help halt or reverse the rising T2DM prevalence (noting the example presented for obesity in see Additional file 1: Figure S5), but there is uncertainty over whether such rapid scale-up of reductions in risk factors are feasible. In reviewing the literature, there does not appear yet to be relevant examples of wide scale population-based interventions that have demonstrated substantial impact. It is also evident that efforts to tackle obesity cannot be limited to community awareness programs designed to improve knowledge and encourage personal responsibility, as it has been the case in Qatar in recent years.

While halting the growing T2DM prevalence is difficult to accomplish, public health response should be focused on reducing the onset of new T2DM cases, in other words reducing incidence. The results presented here demonstrate that the reductions in risk factors, particularly obesity, can lead to large reductions in incidence. Despite the clear difficulties, existing evidence suggests that some reductions may be feasible [10–15]. As demonstrated by the Finnish, Chinese, and American diabetes prevention studies, behavioral lifestyle changes were feasible and led to weight loss resulting in as much as a 40% reduction in T2DM incidence [10–12]. Further to these approaches, fiscal and legislative strategies, such as taxation, marketing restrictions, improved food labeling, and reductions in portion sizes, could be promising in reducing these risk factors [27, 28].

The French EPODE program (Ensemble, Prevenons l’Obésité des Enfants) is an example of a community-based multi-faceted intervention strategy that turned out to be effective with 9% reduction between 2005 and 2009 in overweight and obesity among school children [29]. Mexico’s 10% tax on sugar-sweetened beverages is another example of a successful intervention where nearly 190,000 new T2DM cases could be prevented over the next 10 years [28]. The NOURISHING framework is yet a comprehensive policy package (that is applicable globally) to promote healthier eating and prevent obesity [30, 31]. It recognizes that policy actions are needed within the food environment, food system, and behavior change communication to reduce obesity and intake of unhealthy diets. Though the actual impact of the latter strategy (or similar strategies) is still not quantified, this type of strategies is having increasingly more global attention [32].

There is undoubtedly a need to multiply the current efforts on education and counseling on behavioral change to control obesity and T2DM in Qatar. However, governmental support structures should widen their reach to target the overall food environment. In particular, the abundance and ease of access to calorie-dense unhealthy food, subjecting the consumer to market control, have had their share in fueling the development of unhealthy dietary habits. Controlling the unhealthy environmental triggers can only be achieved by robust policies such as food taxation and restrictive legislation imposed on food markets to gradually shape a healthier food culture.

Considerations and limitations may have affected our results. Our results may depend on the type of mathematical model used and can be affected by the availability of representative epidemiological and demographic input data. Since we explored hypothetical scenarios, we did not conduct uncertainty analyses. We assumed specific parameter values and did not explore variability in these parameters. Having said so, the uncertainty interval of the impact of the interventions is likely to be narrow, in view of the earlier uncertainty analysis, we conducted for the projections of T2DM in Qatar [20]. In most analyses, we assumed a fixed rate of reduction in risk factors, but the rate of reduction may vary from 1 year to another in actual implementation. However, we examined the impact of using variable scale-up rates for the case of obesity reduction, and the results affirmed our findings (see Additional file 1: Figure S5).

While we parameterized our model using relevant and current epidemiological input data, the availability of repeated rounds of nationally representative population-based surveys may have improved the model’s ability to make long-term predictions. Only one round of nationally representative survey, the 2012 STEPwise survey [33] was available to use as input for our model. The survey included 2496 Qataris aged 18–64 years old and collected data on DM prevalence using fasting capillary blood glucose testing, while the prevalence of smoking and physical inactivity was estimated based on self-report [33]. The definition of obesity in the survey and in our study was uniform across all age groups, but there is evidence suggesting that the cutoff for obesity might vary across age groups and ethnicity [34].

We focused strictly on reducing obesity in the population, but this may underestimate the impact on T2DM incidence of small reductions in BMI—reductions that do not cross the obesity threshold, but still can reduce T2DM incidence [10–12]. In the analyses examining the impact of physical activity as an intervention, we only factored the direct effect of physical activity on T2DM incidence, which also may underestimate the impact, if this intervention will also impact T2DM incidence indirectly by reducing obesity. However, most studies have identified only small effects of physical activity on weight change [35]. Our study was focused on T2DM, but the reduction in the risk factors would also lower the incidence of other serious morbidities. While the reductions in smoking and physical inactivity affected T2DM incidence rather modestly, such reductions may have had a large impact on reducing simultaneously other NCD morbidities, such as cancer and cardiovascular diseases.

## Conclusions

In conclusion, halting or reversing the expanding T2DM epidemic in Qatar, as elsewhere, is very challenging. However, major reductions in T2DM incidence could be accomplished by targeting the key risk factors driving T2DM incidence, in particular obesity, the leading driver of incidence. Nearly 40% of T2DM incidence can be prevented in 2050 by reducing obesity prevalence by 50%. Even modest and more realistic reductions in obesity would have a sizeable impact on T2DM incidence. These findings affirm the relevance of the concept of population-level *risk factor intervention-for-prevention*, as a critical and indispensable approach to addressing the rising disease burden of T2DM in this nation.

## Supplementary information

**Additional file 1.** Text S1, Figures S1–S6, Tables S1–S2. Supplementary materials.

## Data Availability

Data are available in the cited literature, main manuscript, and appendix. The codes programmed in MATLAB can be obtained by contacting the authors.

## Abbreviations

EPODE: Ensemble, Prevenons l’Obésité des Enfants
NCD: Noncommunicable diseases
T2DM: Type 2 diabetes mellitus
WHO: World Health Organization
