# Supplementary material for: Preventing type 2 diabetes mellitus in Qatar by reducing obesity, smoking, and physical inactivity: mathematical modeling analyses
Source: Popul Health Metr. 2019 Dec 30;17:20. doi: 10.1186/s12963-019-0200-1 (PMC6937668; doi:10.1186/s12963-019-0200-1)
Supplement: Supplementary file 1 — Additional file 1. Text S1, Figures S1–S6, Tables S1–S2. Supplementary materials. [file 12963_2019_200_MOESM1_ESM.docx]

**Preventing Type 2 Diabetes Mellitus in Qatar by Reducing Obesity, Smoking, and Physical Inactivity: Mathematical Modeling Analyses**

**Appendix**

Susanne F. Awad,^1,2^ Martin O’Flaherty,^3^ Katie G. El-Nahas,^4^ Abdulla O. Al-Hamaq,^4^ Julia A.Critchley,^2^ and Laith J. Abu-Raddad^1,5,6^

^1^Infectious Disease Epidemiology Group, Weill Cornell Medicine - Qatar, Doha, Qatar

^2^Population Health Research Institute, St George’s, University of London, London, UK

^3^Division of Public Health, University of Liverpool, Liverpool, UK

^4^Qatar Diabetes Association, Doha, Qatar

^5^Department of Healthcare Policy and Research, Weill Cornell Medicine, Cornell University, New York, New York, USA

^6^College of Health and Life Sciences, Hamad bin Khalifa University, Doha, Qatar

**Text S1 - Further Details on the Methods**

1. **Description and mathematical equations of the type 2 diabetes mellitus model**

We extended a recently developed type 2 diabetes mellitus (T2DM) mathematical model to investigate the impact of different modelled “what if” intervention scenarios on the T2DM burden in a population (1). The natural history in the T2DM model was described by the general states of 1) healthy, 2) obese, 3) smoker, 4) physically inactive, 5) obese and smoker, 6) obese and physically inactive, 7) smoker and physically inactive, 8) obese, smoker, and physically inactive, 9) T2DM disease (including all risk factor states), and 10) intervention status (including also all risk factor states; Figure S1). The model was programmed in MATLAB version 2015a (2).

Details of the original T2DM model can be found in Awad et al. (1). Briefly, the model is a population-based deterministic compartmental model that accounts for the epidemiological dynamics of T2DM. The population size was incorporated according to population growth in the considered population and divided based on sex and age (index a) where the flow from one age group to the next was dictated by the rate $\varsigma$. We modelled 20 categories of 5-year age groups, of which the first three were children (ages 0–14).

Susceptible individuals remained in the model as such until they progressed to T2DM disease or died of natural causes. Individuals with T2DM remained living with diabetes (i.e. there is no remission), or died of natural or disease-related causes. Individuals in the population with none of the risk factors introduced in the model (whether susceptible, had T2DM, or were in the intervention groups) could develop a risk factor. Individuals in the population with one risk factor could develop the second risk factor, or reverse to a state with none of the risk factors. Individuals in the population with two risk factors could develop the third risk factor, or reverse to only one of the risk factors. Individuals in the population with three risk factors could reverse one of the risk factors. Individuals moved to the prevention intervention states at a fixed rate $\vartheta$. Definitions of all symbols in the equations of the model can be found in Tables S1 and S2.

1. **No intervention branch of the equations**

*A.1.1 Susceptible population with up to one risk factor (for 0–4 years old)*

The susceptible population in age group 0-4 years old was assumed born not to have T2DM nor T2DM-related risk factors and remained in the “healthy” state.

*A.2.1 Susceptible population with up to one risk factor (for >4 years old)*

*A.2.2 Susceptible population with overlap of more than one risk factor (for >4 years old)*

*A.2.3 Populations with T2DM with up to one risk factor (for >4 years old)*

*A.2.4 Populations with T2DM with more than one risk factor (for >4 years old)*

1. **Intervention branch of the equations**

Interventions were assumed administered to only those >4 years old.

*B.1.1 Susceptible population with up to one risk factor*

*B.1.2 Susceptible population with overlap of more than one risk factor*

*B.1.3 Populations with T2DM with up to one risk factor (for >4 years old)*

*B.1.4 Populations with T2DM with more than one risk factor (for >4 years old)*

The following assumptions were made for simplicity and in absence of specific-data on them in the specifically-modeled population, that of Qataris in Qatar:

1. The rate in which individuals become obese in the population was assumed to be independent of their health status.
2. The rate in which individuals become smokers in the population was assumed to be independent of their health status.
3. The rate in which individuals become physically inactive in the population was assumed to be independent of their health status.
4. The rate in which individuals become non-obese in the population was assumed to be independent of their health status.
5. The rate in which individuals quit smoking in the population was assumed to be independent of their health state.
6. The rate in which individuals leave the physically inactive state in the population was assumed to be independent of their health status.
7. All rates in the model were assumed to be independent of the intervention status of the individuals in the population.

**TABLES:**

**Table S1. Definitions of the symbols in the equations of the type 2 diabetes mellitus (T2DM) age-structured mathematical model**

| Symbol^*^ | Definition |
| --- | --- |
| $H_{a}$ | “Healthy” T2DM-susceptible population (do not have T2DM nor T2DM-related risk factors) |
| $O_{a}$ | T2DM-susceptible but obese population^#^ |
| $S_{a}$ | T2DM-susceptible but smoker population |
| $F_{a}$ | T2DM-susceptible but physically inactive population |
| $OS_{a}$ | T2DM-susceptible but obese and smoker population |
| $OF_{a}$ | T2DM-susceptible but obese and physically inactive population |
| $SF_{a}$ | T2DM-susceptible but smoker and physically inactive population |
| $OSF_{a}$ | T2DM-susceptible but obese, smoker, and physically inactive population |
| $D_{\iota}$ | Populations with T2DM where the index $\iota$ marks the risk factor status; $\iota=H, O,\text{ }S, \text{ }F, \text{ }OS, \text{ }OF, \text{ }SF,\text{ }OSF$ |
| $N$ | Total population size |
| $\varsigma$ | Transition rate from one age group to the next age group |
| $\lambda_{a}$ | T2DM-disease progression rate |
| $\mu_{a}$ | Natural death rate |
| $cf_{a}$ | T2DM-related death rate |
| $\vartheta_{\iota,a}$ | Intervention scale-up rate. Index $\iota$ marks the risk factor status; $\iota=H, O,\text{ }S, \text{ }F, \text{ }OS, \text{ }OF, \text{ }SF,\text{ }OSF$ |
| *IE* | The reduction in the risk of developing T2DM due to increase in physical activity |
| $RR_{O}$ | Relative risk of developing T2DM if obese |
| $RR_{S}$ | Relative risk of developing T2DM if smoker |
| $RR_{F}$ | Relative risk of developing T2DM if physical inactive |
| $RR_{OS}$ | Relative risk of developing T2DM if obese and smoker |
| $RR_{OF}$ | Relative risk of developing T2DM if obese and physical inactive |
| $RR_{SF}$ | Relative risk of developing T2DM if smoker and physical inactive |
| $RR_{OSF}$ | Relative risk of developing T2DM if obese, smoker, and physical inactive |
| $\alpha_{a}$, $\beta_{a}$, $\Im_{a}$ | Transition rates from “healthy” with none of the risk factors to one of the risk factors; i.e. become obese ($O$), smoker ($S$), or physically inactive ($F$) |
| $\nu_{a}$, $\eta_{a}$, $\chi_{a}$,$\omega_{a}$, $\xi_{a}$,$\psi_{a}$ | Transition rates from having one of the risk factors to having two risk factors (i.e. becomes $OS$, $OF$, or $SF$) |
| $\sigma_{a}$, $\delta_{a}$, | Transition rates from having one of the risk factors to being “healthy” with none of the risk factors |
| $\kappa_{a}$, $Є_{a}$, $\Omega_{a}$ | Transition rates from having two of the risk factors to having three of the risk factors |
| $\varepsilon_{a}$, $\theta_{a}$, $\gamma_{a}$, $\pi_{a}$, , $\rho_{a}$ | Transition rates from having two of the risk factors to having one of the risk factors |
| , $\iota_{a}$, $\upsilon_{a}$ | Transition rates from having three of the risk factors to having two of the risk factors |
| $m_{a}$, $n_{a}$, $o_{a}$ | Transition rates from being with T2DM with none of the risk factors to having one of risk factors (i.e. becomes $D_{O}$, $D_{S}$, or$D_{F}$) |
| $e_{a}$, $j_{a}$, $f_{a}$, $l_{a}$, $g_{a}$, $h_{a}$ | Transition rates from being with T2DM with one of the risk factors to being with T2DM with two of the risk factors (i.e. becomes $D_{OS}$, $D_{OF}$, $D_{SF}$) |
| $r_{a}$, $q_{a}$, $p_{a}$ | Transition rates from being with T2DM with one of the risk factors to being with T2DM with none of the risk factors |
| $x_{a}$, $w_{a}$, $v_{a}$ | Transition rates from being with T2DM with two of the risk factors to being with T2DM with three of the risk factors (i.e. becomes $D_{OSF}$) |
| $a_{a}$, $i_{a}$, $b_{a}$, $k_{a}$, $c_{a}$, $d_{a}$ | Transition rates from being with T2DM with two of the risk factors to being with T2DM with one of the risk factors |
| $s_{a}$, $t_{a}$, $u_{a}$ | Transition rates from being with T2DM with three of the risk factors to being with T2DM with two of the risk factors |

^*^ Much of these symbols are repeated in the equations, but with *I* at the front, or as an index, indicating the intervention branch of each state in the T2DM age-structured model.

*^#^* Defined as BMI>30 kg per m^2^ (3).

**Table S2. Model assumptions in terms of parameter values.**

| Assumption | Age group | Parameter value (95% CI) | | Reference |
| --- | --- | --- | --- | --- |
|  |  | Male | Female |  |
| Number of age compartments in the model (each for 5 years; *a*) | - | 20 | 20 | - |
| Relative risk of developing T2DM*^#^* if obese ($RR_{O}$) | All | 6.48 (5.17–8.13) | 8.38 (5.46–12.85) | (4) |
| Relative risk of developing T2DM if current smoker ($RR_{S}$) | All | 1.42 (1.34–1.50) | 1.33 (1.26–1.41) | (5) |
| Relative risk of developing T2DM if physically inactive ($RR_{F}$) | 15–69  70–79  ≥80 | 1.45 (1.37–1.54)  1.32 (1.25–1.40)  1.20 (1.14–1.28) | 1.45 (1.37–1.54)  1.32 (1.25–1.40)  1.20 (1.14–1.28) | (6) |
| Relative risk of developing T2DM if obese and smoker ($RR_{OS}$) | All | 9.20 (6.93–12.20) | 11.15 (6.88–18.12) | Calculated based on (4, 5) |
| Relative risk of developing T2DM if obese and physically inactive ($RR_{OF}$) | 15–69  70–79  ≥80 | 9.40 (7.08–12.52)  8.55 (6.46–11.38)  7.78 (5.89–10.41) | 12.15 (7.48–19.79)  11.06 (6.83–18.12)  10.06 (6.22–16.45) | Calculated based on (4, 6) |
| Relative risk of developing T2DM if smoker and physically inactive ($RR_{SF}$) | 15–69  70–79  ≥80 | 2.06 (1.84–2.37)  1.87 (1.68–2.17)  1.70 (1.53–1.97) | 1.93(1.73–2.17)  1.76 (1.58–1.99)  1.60 (1.44–1.80) | Calculated based on (5, 6) |
| Relative risk of developing T2DM if obese, smoker, and physically inactive ($RR_{OSF}$) | 15–69  70–79  ≥80 | 13.34 (9.49–19.28)  12.15 (8.66–17.65)  11.04 (7.90–16.03) | 16.16 (9.43–27.90)  14.71 (8.60–25.55)  13.37 (7.84–23.19) | Calculated based on (4-6) |
| Relative risk of mortality in persons with T2DM compared to the general population ($RR_{M}$) | 20–29  30–39  40–49  50–59  60–69  70–79+ | 3.70  3.30  1.95  1.65  1.62  1.40 | 5.95  5.61  3.41  2.73  2.08  1.78 | (7, 8) |
| Relative risk for developing T2DM with vigorous physical activity | All | 0.61 | 0.61 | (9) |
| Relative risk for developing T2DM with low-intensity physical activity | All | 0.66 | 0.66 | (9) |
| Relative risk for developing T2DM with moderate physical activity | All | 0.68 | 0.68 | (9) |
| Relative risk for developing T2DM with leisure-time physical activity | All | 0.74 | 0.74 | (9) |
| Relative risk for developing T2DM with walking | All | 0.85 | 0.85 | (9) |

*^#^*T2DM: Type 2 diabetes mellitus

**ADDITIONAL FIGURES**


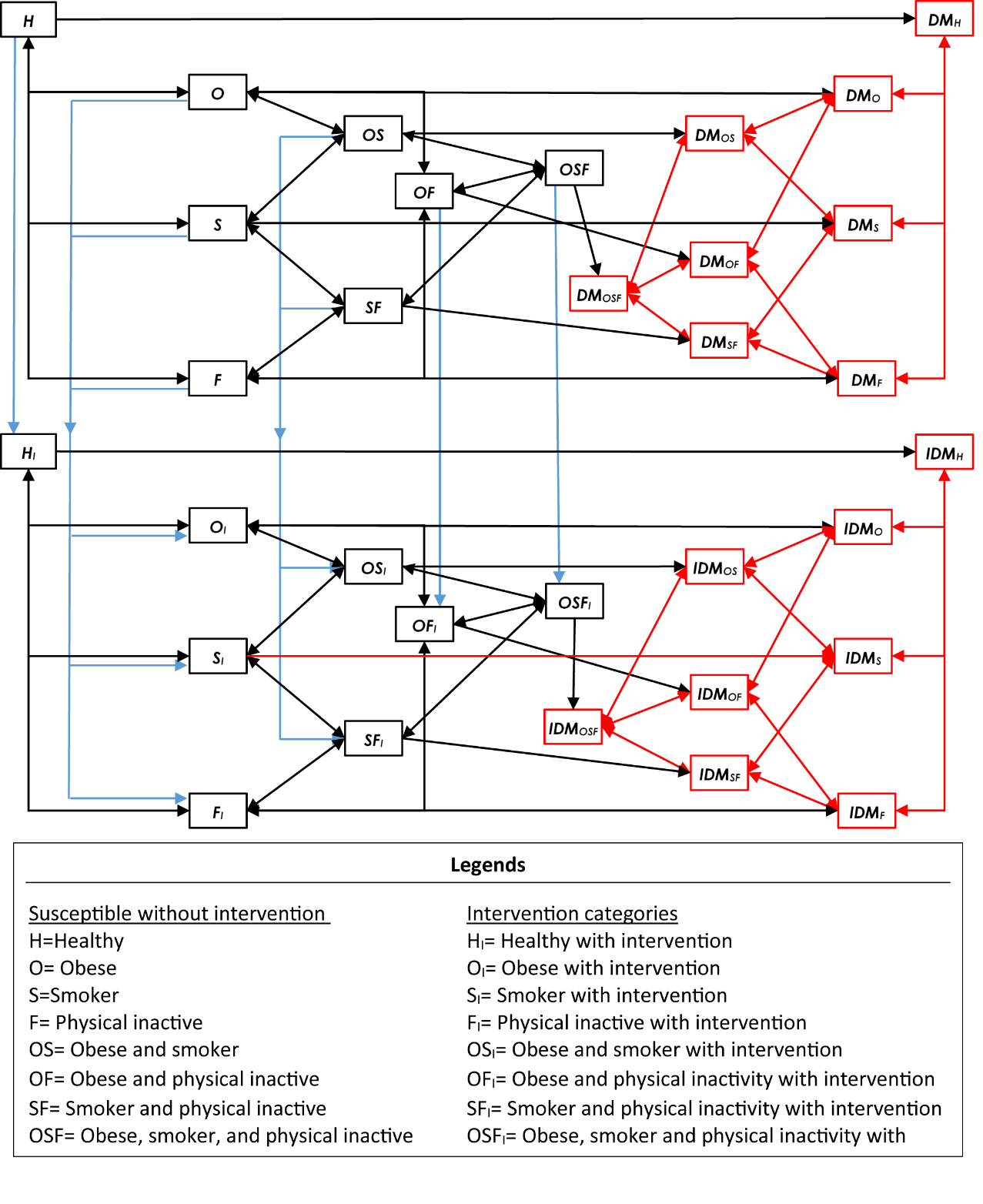

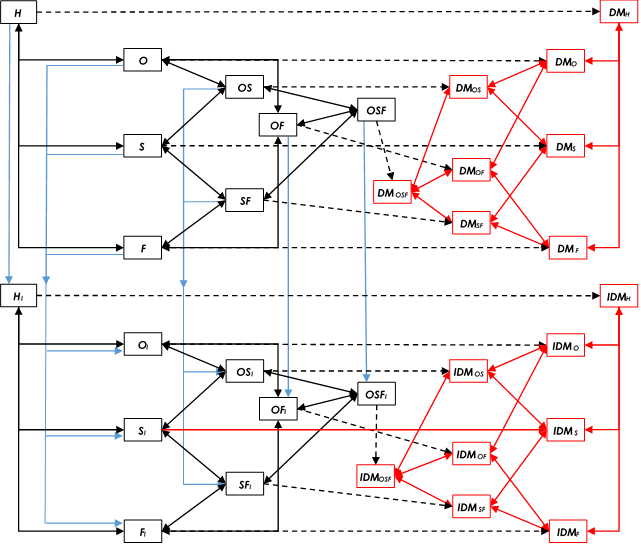


**Legends**

Without intervention categories Intervention (physical activity) categories

**H**=Healthy **H_I_**=Healthy with intervention

**O**=Obese **O_I_**= Obese with intervention

**S**=Smoker **S_I_**= Smoker with intervention

**F**=Physically inactive **F_I_**= Physically inactive with intervention

**OS**=Obese and smoker **OS_I_**= Obese and smoker with intervention

**OF**=Obese and physically inactive **OF_I_**= Obese and physically inactive with intervention

**SF**=Smoker and physically inactive **SF_I_**= Smoker and physically inactive with intervention

**OSF**=Obese, smoker, and physically inactive **OSF_I_**= Obese, smoker, and physically inactive with intervention

**DM_X_**=Disease based on health state X **IDM_X_**= Disease based on health state X with intervention

**Figure S1. Conceptual framework for the epidemiological dynamics of type 2 diabetes mellitus (T2DM) in the population, the effects of T2DM-related risk factors on T2DM, and the effects of a health prevention intervention (physical activity) on T2DM.** The black solid lines indicate the transitions within the susceptible states (“healthy”, “obese”, “smoker”, and “physically inactive” states, and their overlapping compartments). The black dashed lines indicate the transitions from each one of the susceptible states to the T2DM disease states. The red lines indicate the transitions within the T2DM disease states. The blue lines indicate the transitions from states without the intervention to states with the intervention.

**
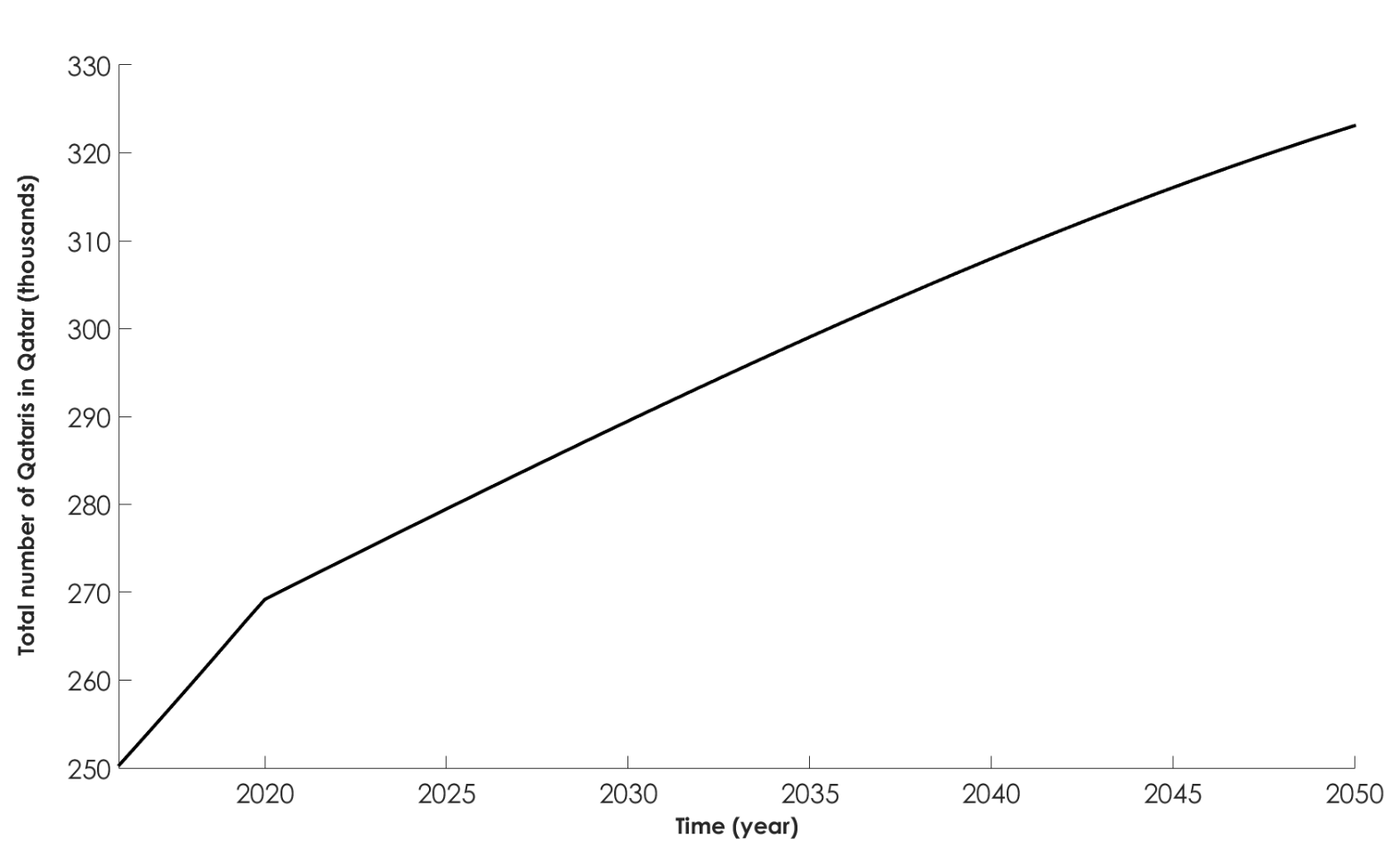
**

**Figure S2. Model-projected demographics of the Qatari population between 2016 and 2050.**

**
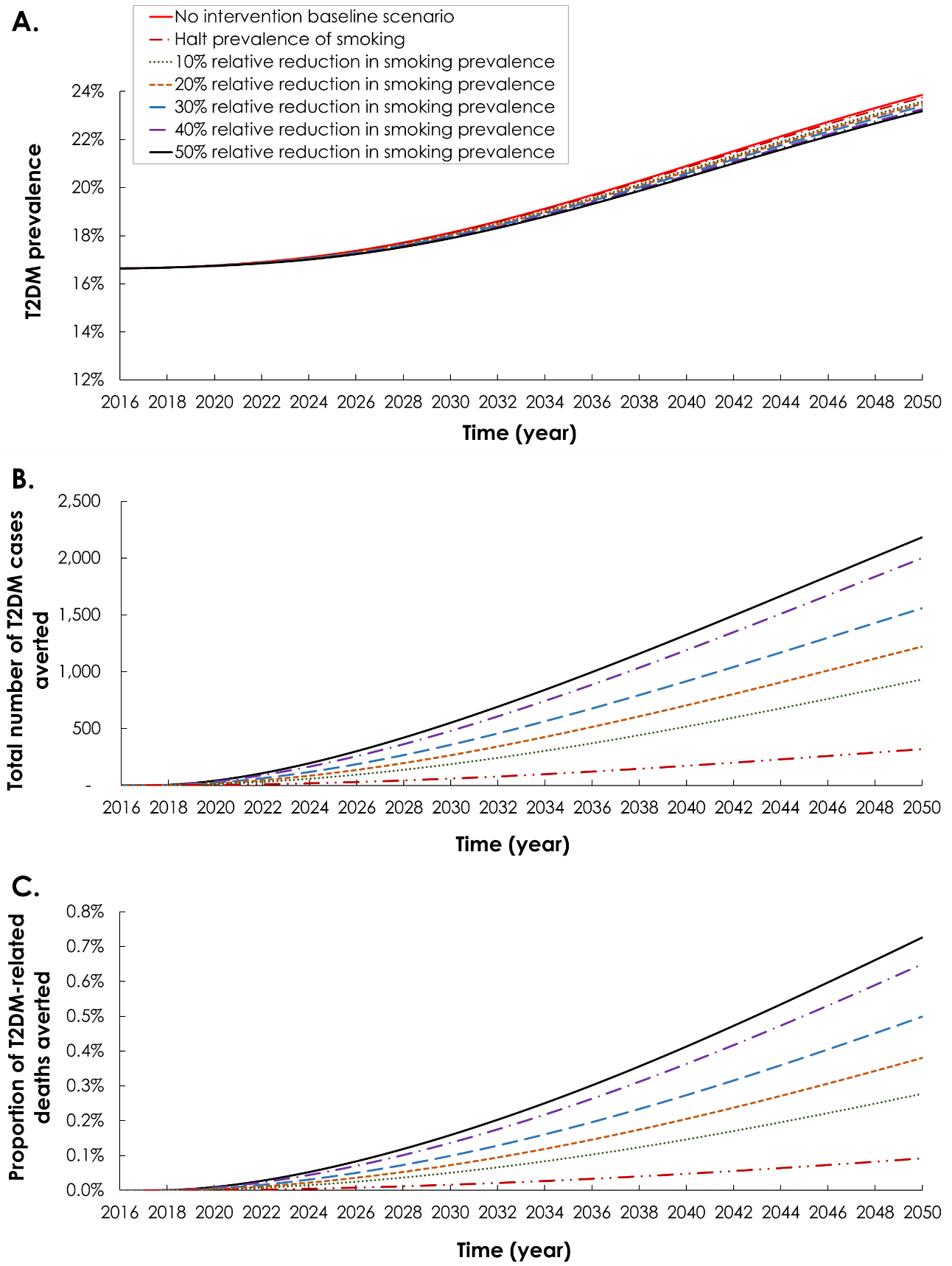
**

**Figure S3: Epidemiologic impact of reducing the prevalence of smoking among Qataris 15-64 years of age.** The figure shows the **A)** projection of type 2 diabetes mellitus (T2DM) prevalence, **B)** number of T2DM cases averted, and **C)** proportion of T2DM-related deaths averted. The red solid curve in panel A shows the prediction of T2DM prevalence in the no intervention scenario (baseline scenario).

**
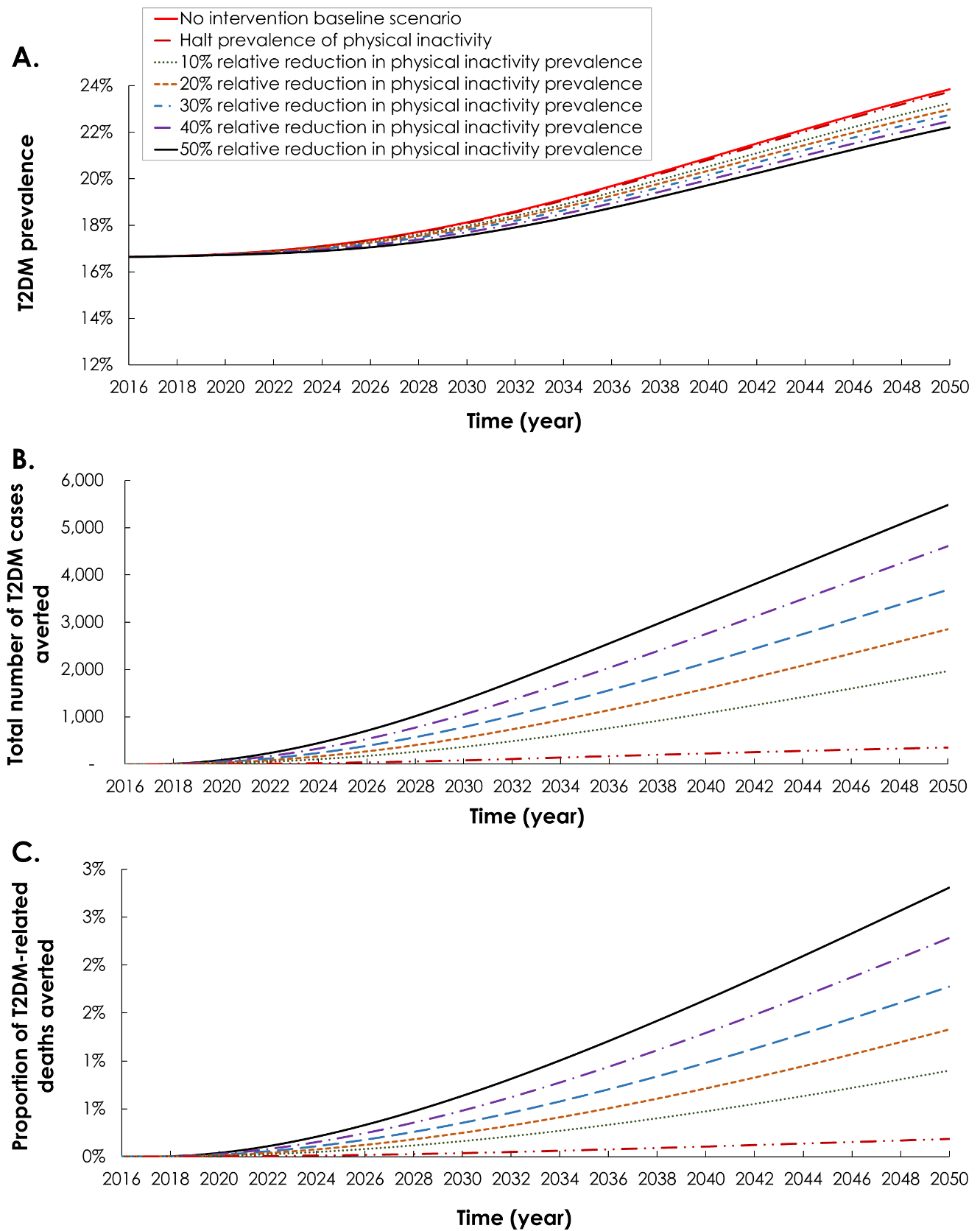
**

**Figure S4: Epidemiologic impact of reducing the prevalence of physical inactivity among Qataris 15-64 years of age.** The figure shows the **A)** projection of type 2 diabetes mellitus (T2DM) prevalence, **B)** number of T2DM cases averted, and **C)** proportion of T2DM-related deaths averted. The red solid curve in panel A shows the prediction of T2DM prevalence in the no intervention scenario (baseline scenario).


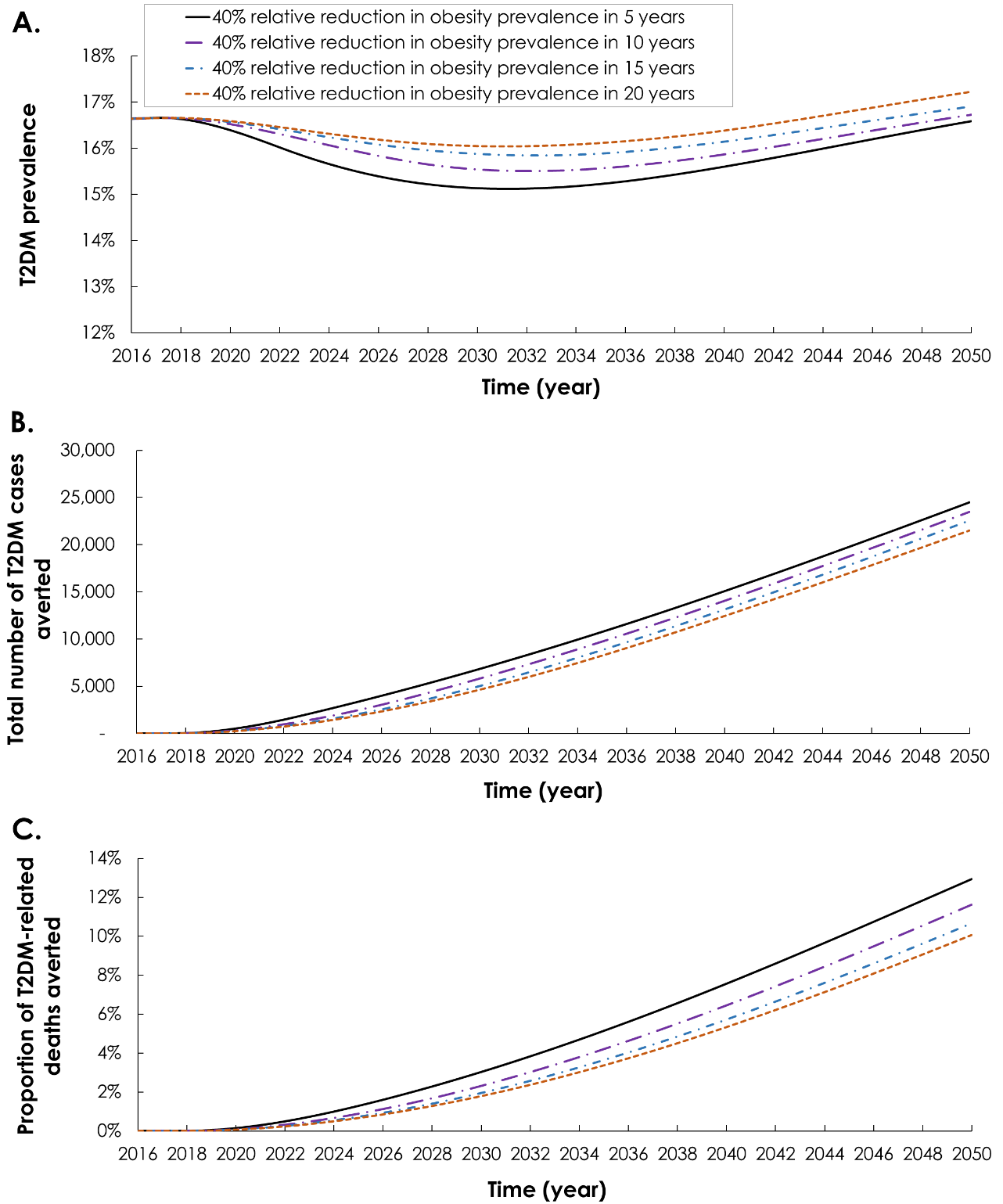


**Figure S5. Additional analysis assessing the impact on type 2 diabetes mellitus (T2DM) cases and deaths of reducing the prevalence of obesity by 40%, but at variable scale-up durations.** The prevalence of obesity was then maintained between 2032 and 2050.


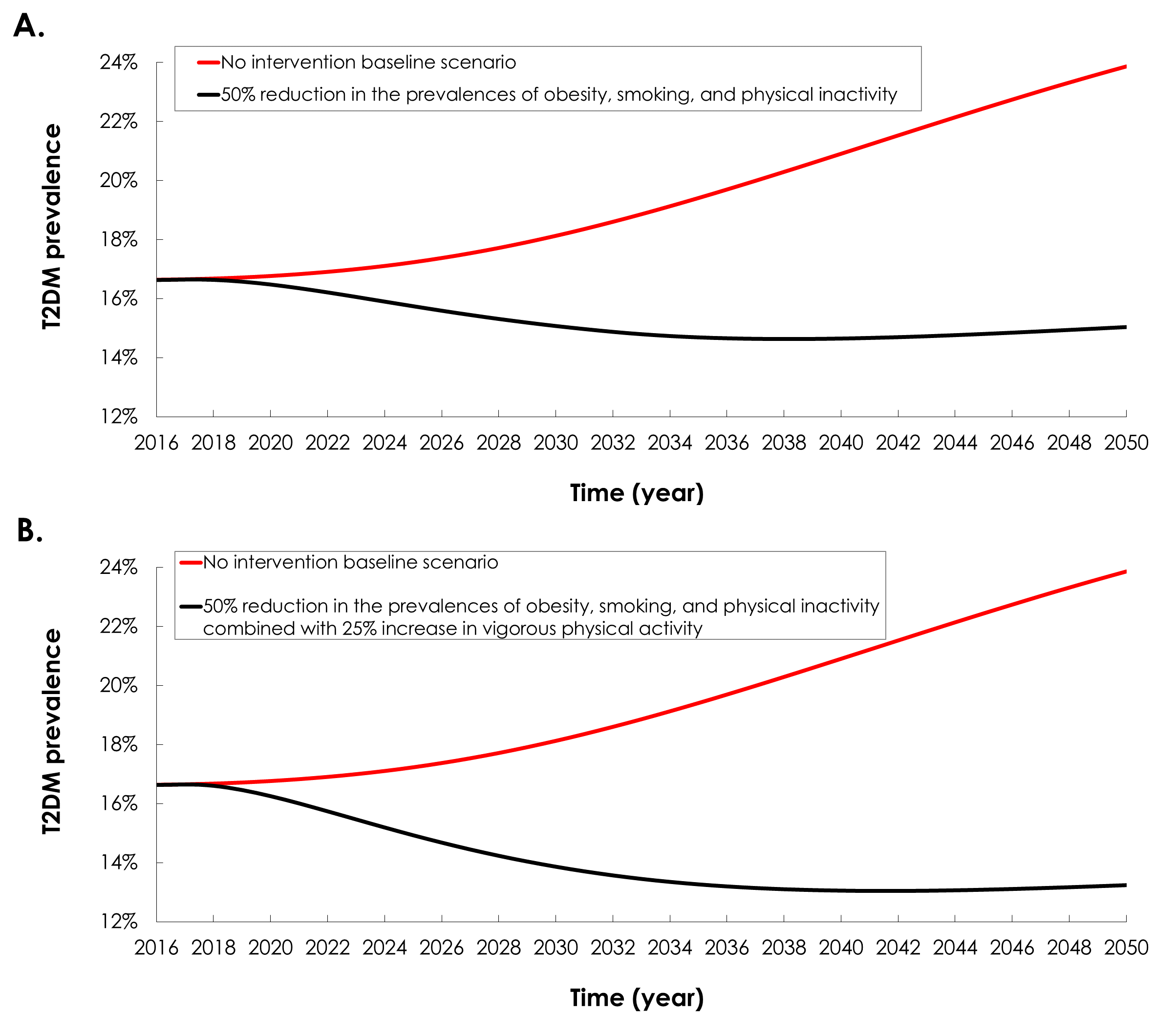


**Figure S6. Analysis assessing the impact on type 2 diabetes mellitus (T2DM) prevalence of A) simultaneously reducing the prevalence of obesity, smoking, and physical inactivity by 50%, and B) simultaneously reducing the prevalence of obesity, smoking, and physical inactivity by 50% combined with a 25% increase in vigorous physical activity.**

**REFERENCES**

1. Awad SF, O'Flaherty M, Critchley J, Abu-Raddad LJ. Forecasting the burden of type 2 diabetes mellitus in Qatar to 2050: A novel modeling approach. Diabetes research and clinical practice. 2018;137:100-8.

2. The MathWorks, Inc. MATLAB. The language of technical computing. 8.5.0.197613 (R2015a). Natick, MA, USA: ed: The MathWorks, Inc.; 2015.

3. World Health Organization. Obesity and overweight factsheet (available at: <http://www.who.int/mediacentre/factsheets/fs311/en/>) 2015 [

4. Abdullah A, Peeters A, de Courten M, Stoelwinder J. The magnitude of association between overweight and obesity and the risk of diabetes: a meta-analysis of prospective cohort studies. Diabetes research and clinical practice. 2010;89(3):309-19.

5. Pan A, Wang Y, Talaei M, Hu FB, Wu T. Relation of active, passive, and quitting smoking with incident type 2 diabetes: a systematic review and meta-analysis. The lancet Diabetes & endocrinology. 2015;3(12):958-67.

6. Fiona C. Bull, Timothy P. Armstrong, Tracy Dixon SH, Andrea Neiman, Pratt M. Comparative Quantification of Health Risks. Global and Regional Burden of Disease Attribution to Selected Major Risk Factors. Chapter 10: Physical Inactivity. (available at: <http://www.who.int/publications/cra/chapters/volume1/0729-0882.pdf?ua=1>). World Health Organization; 2004.

7. Nakagami T, Group DS. Hyperglycaemia and mortality from all causes and from cardiovascular disease in five populations of Asian origin. Diabetologia. 2004;47(3):385-94.

8. International Diabetes Federation. IDF Diabetes Atlas. 3th edition. Brussels, Belgium (available at: <https://www.idf.org/sites/default/files/Diabetes-Atlas-3rd-edition.pdf>; accessed on December 2015). 2006.

9. Aune D, Norat T, Leitzmann M, Tonstad S, Vatten LJ. Physical activity and the risk of type 2 diabetes: a systematic review and dose-response meta-analysis. Eur J Epidemiol. 2015;30(7):529-42.
